# Neural correlates of affective stimulus evaluation: a case-by-case analysis

**DOI:** 10.1093/scan/nsab095

**Published:** 2021-08-06

**Authors:** Harald T Schupp, Ursula Kirmse

**Affiliations:** General and Biological Psychology, Department of Psychology, University of Konstanz, Konstanz 78457, Germany; Centre for the Advanced Study of Collective Behaviour, University of Konstanz, Konstanz 78457, Germany; General and Biological Psychology, Department of Psychology, University of Konstanz, Konstanz 78457, Germany

**Keywords:** emotion, attention, ERP, LPP, case by case

## Abstract

A recent study provided first evidence that neural correlates of affective stimulus evaluation, that is, the early posterior negativity (EPN) and late positive potential (LPP), can be assessed at the individual case level. Expanding the case-by-case approach, the main aim of the present study was to explore the process of affective stimulus evaluation within the individual participant with respect to multiple emotional stimulus classes. Toward this end, each participant viewed separate blocks of low- and high-arousing pictures from behavior systems of predator fear, disease avoidance and sexual reproduction. Thirteen out of 16 participants showed larger EPN and LPP amplitudes for higher- than lower-arousing stimuli for all three behavior systems. Furthermore, rather than indicating a general lack of emotional modulation, cases of non-significant EPN (*N* = 3) and LPP (*N* = 2) tests in individual participants appeared to be specific to a single emotion category. Overall, assessing the emotional modulation of the EPN and LPP across multiple behavior systems strengthens the case-by-case approach regarding an effect that is ‘common to all’ as well as by differentiating non-significant effects within individuals in terms of a content-specific or general phenomenon. Implications for revealing a general principle of emotion functioning and biomarker development are discussed.

## Introduction

Every stimulus that we encounter is evaluated according to its affective significance. An evolutionary perspective suggests that detecting significant stimuli early in the processing stream facilitates adaptive responding by allocating attentional resources to the processing of stimuli (Lang *et al.*, 1997; Öhman *et al.*, 2000). Numerous studies used event-related potentials (ERPs) to reveal the selective processing of emotionally significant stimuli. Specifically, the processing of high-arousing emotional (pleasant and unpleasant) images compared to low-arousing control images is associated with a negative-going difference potential over temporo-occipital sensor regions around 150–300 ms post-stimulus, referred to as early posterior negativity (EPN). Subsequently, a positive potential difference over centro-parietal regions is observed between 350 and 700 ms denoted as late positive potential (LPP) (Cuthbert *et al.*, 2000; Junghöfer *et al.*, 2001; Schupp *et al.*, 2006a).

Most previous research demonstrated these effects based on group analysis (Schupp *et al.*, 2006a; Hajcak *et al.*, 2010; Schindler and Bublatzky, 2020). Consequently, the findings refer to a ‘hypothetical person’, i.e. the group mean average. A stronger support for demonstrating the selective processing of emotionally significant stimuli is provided by the case-by-case approach (Danziger, 1990; Lamiell, 1998). Serving as a proof of principle, recent research determined the proportion of individual cases showing significantly larger EPN and LPP components to emotionally arousing stimuli (Schupp and Kirmse, 2021). Across three studies, the majority of participants showed significantly larger EPN (92%) and LPP (98%) amplitudes for high- than low-arousing pictures selected from behavior systems of sexual reproduction, disease avoidance and predator fear.

To which extent the case-by-case approach can reveal a general principle related to affective stimulus evaluation depends on subject generality, that is, representativeness of the effect, and generality of variables, that is, demonstrating the effect across emotional stimulus categories (Sidman, 1960). Thus, each case that does not show larger EPN and LPP components for high-arousing rather than low-arousing emotional stimuli jeopardizes the notion that the effect is common to all cases of the research sample. However, the interpretation of a non-significant test result is ambiguous due to the lack of an objective measure to validate it. Specifically, with the current neural measurement techniques, the neural representation coding for emotional stimulus significance is inaccessible. Furthermore, idiosyncratic learning experiences may alter some of the individual’s responses to evolutionary significant stimuli, resulting in true non-significant EPN and LPP findings. Finally, considering notable dissociations among the various emotional response measures, i.e. somatic, autonomic, self-reported and behavioral (Lang, 1978; Miller and Kozak, 1993), the event-related potential (ERP) findings cannot be validated using other emotional response measures. However, the ambiguity of interpreting non-significant cases can be reduced when the process of affective stimulus evaluation is examined within each person for multiple emotion categories. Specifically, a general lack of emotional modulation of the EPN and LPP should be independent of stimulus contents. Conversely, a case showing a non-significant test for a specific stimulus category accompanied by significant emotional modulation effects for other categories may indicate a content-specific rather than a general effect within the individual.

Furthermore, studying the process of affective stimulus evaluation across multiple emotion categories within individuals is also relevant for exploring the potential of the case-by-case approach toward biomarker development and translating basic research to the clinical domain. With the unit of analysis being the individual case, the leap of faith that is needed for making individual inferences based on aggregated group data can be circumvented (Barlow and Nock, 2009; Buckholtz and Faigman, 2014). In this respect, assessing multiple categories of emotional experience allows to explore the individual emotional landscape, identifying exaggerated/blunted responses to selected contents. Furthermore, regarding the development of robust experimental designs to assess the process of affective stimulus evaluation in the individual case, a cross-category comparison allows the stimulus categories to be determined, leading to the most robust effects in the participants comprising the sample.

The main aim of the present study was to determine the emotional modulation of the EPN and LPP in individual cases for multiple emotional stimulus categories. To this end, the participants viewed low- and high-arousing pictures that were selected from three emotional behavior systems, i.e. sexual reproduction, disease avoidance and predator fear. Representativeness and generality of variables were examined by determining the number of participants showing significant EPN and LPP effects within and across behavior systems. The consistency of findings within individuals across the three behavior systems allows to evaluate cases of non-significant EPN and LPP effects in terms of a stimulus-specific effect, i.e. limited to a single behavior system or a generalized phenomenon across different evolutionary-shaped positive and negative behavior systems. In addition, diagnostic performance was also assessed for specificity (observing no effect when no effect is predicted; Woo and Wager, 2015; van der Miesen *et al.*, 2019). This analysis compared left- and right-mirrored picture categories for which no significant EPN and LPP effect is predicted. Furthermore, considering the *P*-value as a graded measure of effect against the null hypothesis (Button *et al.*, 2013; Halsey *et al.*, 2015), the trade-off between sensitivity and specificity was explored as a function of different *P*-criteria.

## Materials and methods

### Participants

Eighteen participants were recruited from the University of Konstanz. Data were discarded for one participant who appeared overtired and exhausted during the experiment and showed visible signs of sleepiness. Another participant was not considered in the final analysis due to a selective loss of acceptable trials in the disease avoidance condition, where the number of trials (129 out of 1200) was unacceptably low (<11%) for single-subject bootstrap analysis. Thus, the final sample included 16 healthy volunteers (8 ♂/8 ♀) with a mean age of 22.0 years (s.d. = 3.9).

All participants had normal or corrected-to-normal vision and were healthy at the time of testing, reporting no history of neurological or psychiatric disorders. The participants received either monetary compensation or course credit for their participation. The ethical committee of the University of Konstanz approved the experimental procedure in accordance with the regulations of the Declaration of Helsinki, and all methods were carried out in full compliance with the approved guidelines. All participants provided informed consent and were debriefed after the experiment.

### Stimuli

Stimulus materials were selected from behavior systems of predator fear, disease avoidance and sexual reproduction (see Schupp and Kirmse, 2021). The stimuli for each behavior system comprised 20 images, of which 10 were high and 10 were low in emotional arousal. For sexual reproduction, the high-arousing category comprised pictures showing couples in explicit erotic postures, while the low-arousing control category contained pictures showing couples in a romantic pose, i.e. hugging or kissing. For disease avoidance, high-arousing stimuli showed bleeding or injured and/or deformed human bodies, mutilation and injury, while the low-arousing control category comprised pictures showing uninjured humans in neutral poses. For predator fear, the high-arousing stimulus category comprised pictures of wild animals in dangerous, threatening poses (e.g. tiger, shark and alligator), while the low-arousing control category included pictures of harmless animals in non-threatening poses (e.g. cat, sheep and lizard). Images were selected from the International Affective Picture System (IAPS; Lang *et al.*, 2008) and public domain sources. IAPS pictures 4610 and 4658, 3051 and 2200, and 1301 and 1500 show representative exemplars for the three behavior systems. The pictures for the high- and low-arousing stimulus categories within each behavioral system were selected to be similar in overall composition. All images were standardized with respect to brightness and contrast in the red, green and blue channels. Spatial frequencies for picture categories were analyzed using shared code from Delplanque *et al.* (2007). *Z*-score transformed coefficients were computed separately for red, green and blue colors and high and low spatial frequencies (cf., Delplanque *et al.*, 2007). Separate analyses of variance for each behavior system and RGB color revealed neither main effects of picture category (high *vs* low), *F*_s_(1,18) ≤ 2.08, *P*_s_ *>* 0.16, nor significant interactions of picture category by spatial frequency (high *vs* low), *F*_s_(1,18) ≤ 3.99, *P*_s_ > 0.06. To control for horizontal eye movements, pictures were presented in both the original and horizontally flipped directions, resulting in 20 pictures per stimulus category and thus 40 pictures overall for each behavior system.

### Experimental design

The experimental design consisted of three experimental blocks, showing images selected from behavior systems of fear predator, disease avoidance and sexual reproduction, respectively. The order of these experimental blocks was permuted across participants. Aside from stimulus materials, parameters of the experimental design were identical across the three blocks. Specifically, in each condition, pictures were displayed for 150 ms, preceded by a fixation cross shown for 100 ms. The inter-trial interval varied from 617 to 967 ms (*M* = 790 ms). Each picture from the stimulus set (*N* = 40 for each behavioral system) was presented 30 times, resulting in 600 trials per picture category and a total of 1200 presentations for each of the three experimental blocks. Within each condition, the pictures from the two stimulus categories were presented in a pseudo-randomized order for each participant. No more than three consecutive presentations of the same picture category were allowed, and transition frequencies between picture categories were controlled. The participants were instructed to simply view the images. Short breaks of 5–8 min for posture adjustment and impedance checks occurred after half of the trials within each experimental block and between experimental blocks. After each experimental block, the participants rated the stimulus materials for each behavioral system according to emotional dimensions of valence and arousal using the Self-Assessment Manikin (Bradley and Lang, 1994). Overall, the experiment lasted approximately 90–100 min.

### EEG data acquisition and analysis

Brain and ocular scalp potentials were measured with a 256-lead geodesic sensor net (HCGSN), filtered online below 100 Hz and sampled at 250 Hz using Netstation acquisition software and EGI amplifiers (Electrical Geodesics Inc., Eugene, OR). Electrode impedance was kept below 40 kΩ, as recommended by EGI guidelines for this type of electroencephalogram (EEG) amplifier. Data were recorded continuously using the vertex sensor as a reference electrode. Using EMEGS software (Peyk *et al.*, 2011), the continuous EEG data were offline filtered using a digital low-pass filter with a half-power cutoff at 40 Hz (Butterworth IIR filter, order 19, stopband: −45 dB at 50 Hz) and a digital high-pass filter with a half-power cutoff at 0.06 Hz (Butterworth IIR filter, order 4, stopband: −18 dB at 0.05 Hz). The data were then corrected for ocular artifacts based on a multiple regression method. Artifact rejection was performed based on an elaborate method for statistical control of artifacts, specifically tailored for the analysis of dense sensor EEG recordings (Junghöfer *et al.*, 2000). The data were re-referenced to an average reference and baseline-adjusted (100 ms pre-stimulus). To ensure equal representation of high- and low-arousing trials in the bootstrap analyses, trial numbers after artifact rejection were equated between condition by keeping the desired number of trials from the condition with more trials based on a vector of randomly permuted integers. Trial numbers for sexual reproduction (*M* = 447.31, s.d. = 47.49), disease avoidance (*M* = 435.75, s.d. = 59.54) and predator fear (*M* = 429.63, s.d. = 68.20) were not significantly different for the three behavior systems, *F*(2,30) = 0.77, *P* = 0.48. Finally, average waveforms were calculated separately for the low- and high-arousing pictures for the sexual reproduction, disease avoidance and predator fear behavior system.

### EEG signal quality

Sufficient EEG signal quality provides the basis for including the participants in group analyses as well as for conducting bootstrap analysis of individual cases. Two approaches were taken to examine the EEG signal quality for each individual case. Both approaches focused on EEG signal quality by calculating the ERP averaged across the sensors comprising the central *vs* posterior cluster (see below).

The first approach allowed a visual comparison of the measured ERP waveform to the ‘(±) reference’ ERP, which removes the ERP signal from the waveform by alternating the polarity of every second trial before averaging (Schimmel, 1967). For both the posterior and central sensor clusters, the comparison of the regular ERP waveform to the (±) reference ERP indicated that the ERPs to stimuli from sexual reproduction, disease avoidance and predator fear represented EEG signals going beyond background noise levels for each individual subject.

A second approach provided quantitative analysis of the EEG signal quality by calculating signal-to-noise confidence intervals (for details, see Parks *et al.*, 2016). Specifically, a bootstrap procedure was used to calculate the ratio of root mean square post- and pre-stimulus activities (expressed logarithmically in decibels) by resampling based on the number of trials from the picture category (low and high arousal) with the fewest number of trials. As recommended, the 90% confidence interval of the resampled signal-to-noise ratios was calculated (Parks *et al.*, 2016). The lower boundary of the signal-to-noise confidence interval of the individual cases, calculated separately for each behavioral system ranged from 12.2 to 27.5 dB (*M* = 18.9, s.d. = 3.74) for the posterior sensor cluster and from 9.7 to 23.9 dB (*M* = 16.7, s.d. = 3.85) for the central sensor cluster (see below), exceeding the 3 dB minimum threshold of signal quality recommended by Parks *et al.* (2016) for each individual case.

### Group analysis

EPN and LPP clusters were a priori defined based on previous research (Schupp and Kirmse, 2021). Accordingly, the EPN was scored as mean activity in a time window from 240 to 300 ms in an occipito-parietal sensor cluster comprising the following sensors: 106, 107, 108, 113, 114, 115, 116, 117, 121, 122, 123, 124, 125, 126, 133, 134, 135, 136, 137, 138, 139, 145, 146, 147, 148, 149, 150, 151, 156, 157, 158, 159, 160, 165, 166, 167, 168, 169, 174, 175 and 176 (see Figure 2). The LPP was scored in a time window from 380 to 480 ms in a centro-parietal cluster including the following sensors: 6, 7, 8, 9, 15, 16, 17, 23, 24, 30, 42, 43, 44, 45, 51, 52, 53, 59, 60, 79, 80, 81, 89, 130, 131, 132, 143, 144, 155, 183, 184, 185, 186, 196, 197, 198, 206, 207, 215 and 257.

**Fig. 1.**
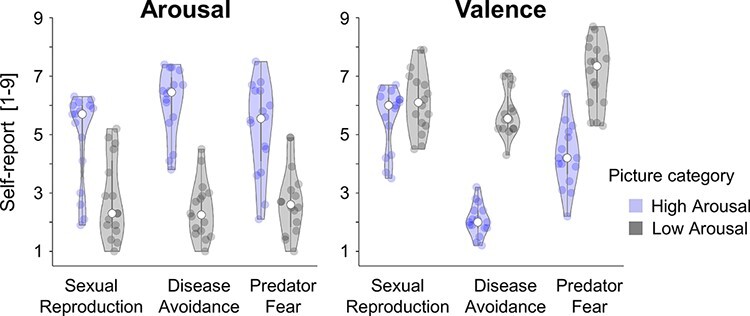
Valence (1—unpleasant to 9—pleasant) and arousal (1—low to 9—high) ratings separately for low- and high-arousing pictures and the three behavior systems, i.e. erotic *vs* romantic couples, mutilations *vs* humans in neutral pose and threatening *vs* harmless animals. The graph was produced using the code provided by Bechtold (2016); the white dot indicates the median value.

**Fig. 2.**
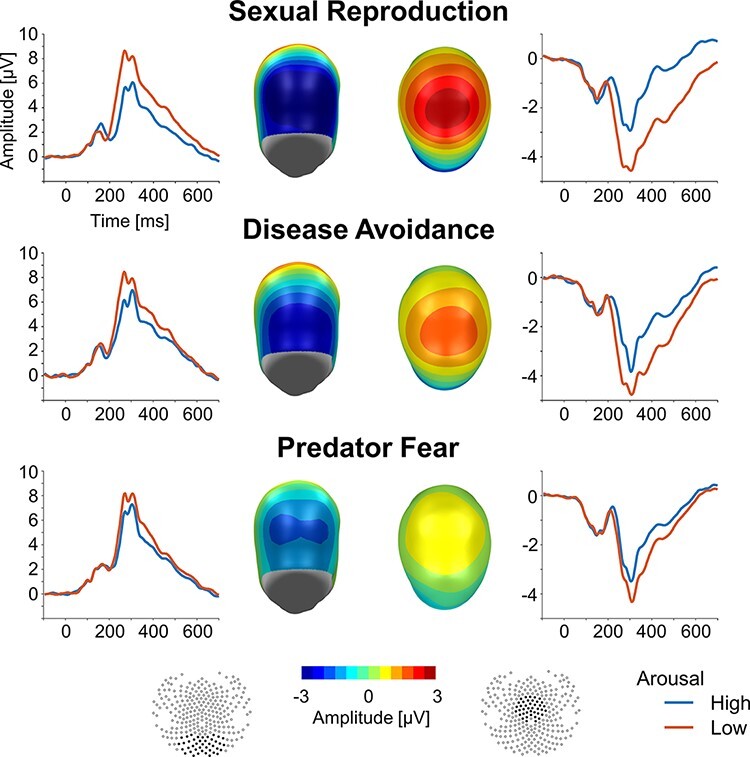
Illustration of ERP waveforms and difference scalp maps (high–low arousal) of the emotional modulation of the EPN (left) and LPP (right). Waveforms represent the average across all sensors of the clusters; sensor positions of the EPN and LPP clusters are shown in black on a model head. The left panels illustrate the EPN effect. While the ERPs of the posterior sensor cluster show positive polarity, the EPN appears as a relative negative shift for high-arousing compared to low-arousing images. Similarly, while the central cluster ERPs have an overall negative polarity, the LPP effect appears as a relative positive shift for high-arousing images (right panels). The scalp maps represent the average difference across a time window from 240 to 300 ms (EPN) and from 380 to 480 ms (LPP), displaying a back (EPN) view and a top (LPP) view of the model head.

Dependent *t*-tests (high- *vs* low-arousing images) were conducted separately for each behavioral system to replicate the findings of larger EPN and LPP amplitudes for emotionally arousing pictures.

### Case-by-case analysis

Single-subject bootstrap analyses were conducted to determine whether individual cases showed significant EPN and LPP differences (high–low arousal) for sexual reproduction, disease avoidance and predator fear (Wasserman and Bockenholt, 1989; Efron and Tibshirani, 1993; Di Nocera and Ferlazzo, 2000; Rosenfeld, 2020). The scoring of the EPN and LPP components for single-subject analysis was based on the same a priori defined sensor clusters of the EPN and LPP used in group analyses. However, the latency of the effect was allowed to vary between participants and emotion categories to acknowledge inter-individual variability in functional brain organization (e.g. Rosenfeld *et al.*, 2006; Oruç *et al.*, 2011). Specifically, the EPN effect was defined to appear within a time window of 150–350 ms and the LPP effect within a time window of 350–750 ms. Within these temporal restrictions, a custom software determined the time window that showed the maximum EPN (negative, spanning 60 ms) and LPP (positive, spanning 100 ms) difference effect for high- *vs* low-arousing pictures for each case. ERP amplitudes for high- and low-arousing pictures were scored in these individual time windows.

For bootstrap analyses, each case’s EPN/LPP mean data were resampled with 50 000 bootstrap repetitions by randomly (re-) assigning a case’s trials to the high- and low-arousing category (drawn with replacement) and calculating the mean difference. Because of the random assignment, the distribution of the 50 000 bootstrap differences represents the empirical probability distribution when no significant differences for the EPN and LPP components are expected. Significance (*P* < 0.05, one-sided) on the individual case level was determined as the proportion of results in the empirical probability distribution that were equal or more extreme than the de facto measured EPN and LPP difference. Results were the same for *P* < 0.05 one-sided and two-sided testing in the main analysis.

### Specificity analysis

Specificity was assessed separately for each behavior system and high- and low-arousing picture categories by splitting each picture set into left- and right-mirrored picture presentations. ERP components were scored with the same parameters as used in the main analysis. As bootstrap analyses were conducted for the two possible directions, i.e. left > right; right > left, and separately for the high and low picture category, there are four times as many tests in the specificity analysis as in the case-by-case analysis. The outcome of the specificity analysis is expressed as the false alarm rate (100-specificity).

### Effects associated with the chosen *P*-level criteria

To reveal how different *P*-criteria affect the number of significant cases, the proportion of significant effects for the single subject main analysis and the specificity analysis are reported for the *P*-level criteria of *P* < 0.05, *P* < 0.025, *P* < 0.01, *P* < 0.001 and *P* < 0.00002, respectively, indicating that less than 2500, 1250, 500, 50 and 0 out of 50 000 randomized calculations yielded an equal or more extreme result.

Furthermore, Cochran-Q tests were used to determine differences in the proportion of significant cases as a function of behavior system and *P*-criteria. Post hoc testing was conducted using Bonferroni correction.

## Results

### Self-report data

Predicted differences in perceived arousal were confirmed for the three behavior systems (see Figure 1). Specifically, erotica compared to romantic pictures, mutilations compared to neutral people and threatening compared to safe animals were evaluated as more arousing, *t*_s_(15) ≥ 5.7, *P* < 0.001. Regarding valence, mutilations compared to neutral people and threatening compared to safe animals were rated as more unpleasant, *t_s_*(15) ≤ −7.2,*P* < 0.001. There was no significant difference for valence regarding erotic and romantic pictures, *t*(15) = −1.5, *P* = 0.16.

### Group analysis

As shown in Figure 2, previous findings on ERP waveforms and emotional modulation of the EPN and LPP components were replicated for all three behavior systems. Specifically, for the EPN, a significant negative difference was observed for sexual reproduction, *M* = −2.80, s.d. = 1.44, *t*(15) = −7.79, *P* < 0.001, Cohen’s *d* = −1.95, disease avoidance, *M* = −2.07, s.d. = 1.19, *t*(15) = −6.97, *P* < 0.001, Cohen’s *d* = −1.74, and predator fear, *M* = −1.30, s.d. = 0.75, *t*(15) = −6.94, *P* < 0.001, Cohen’s *d* = −1.74. Similarly, a significant positive difference was observed for the LPP regarding sexual reproduction, *M* = 2.17, s.d. = 0.86, *t*(15) = 10.1, *P* < 0.001, Cohen’s *d* = 2.51, disease avoidance, *M* = 1.34, s.d. = 0.63, *t*(15) = 8.91, *P* < 0.001, Cohen’s *d* = 2.23, and predator fear, *M* = 0.63, s.d. = 0.32, *t*(15) = 7.82, *P* < 0.001, Cohen’s *d* = 1.95.

### Case-by-case analysis

**Fig. 3.**
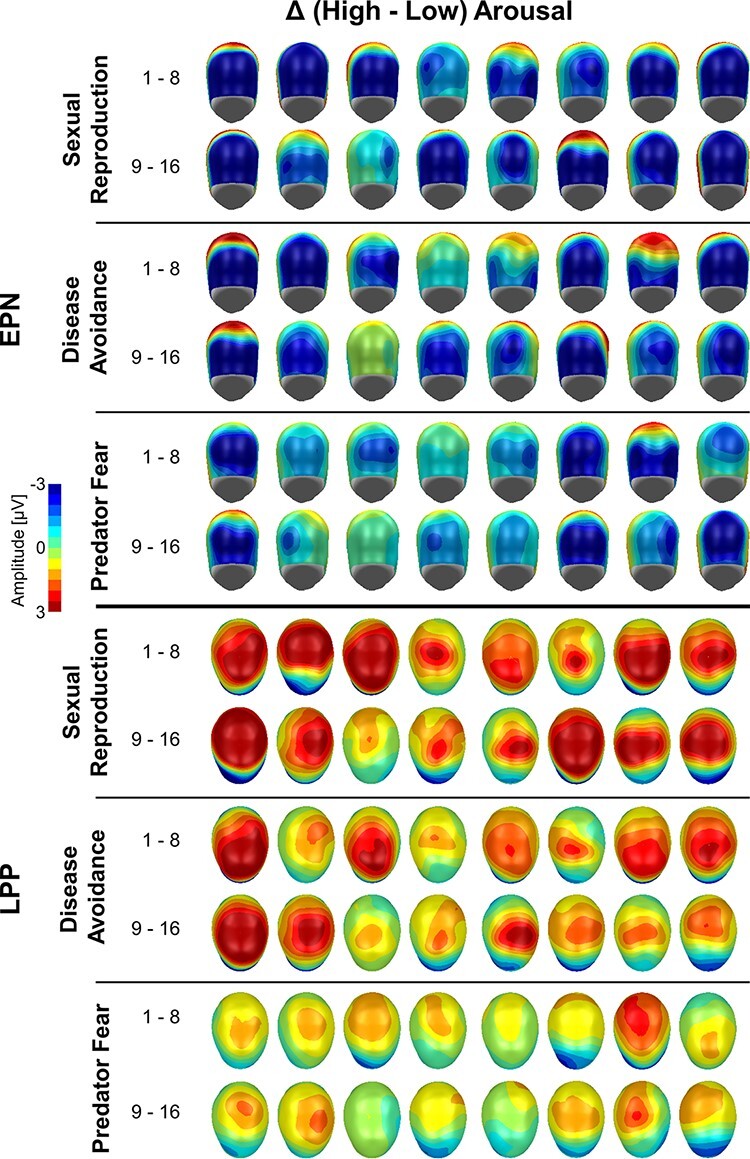
Difference scalp maps (high–low arousal) of the emotional modulation of the EPN and LPP for each individual case and behavior system. Maps show the individual time window of the EPN and LPP difference as selected by the automatic software routine (see the ‘Materials and methods’ section for more details).

Figure 3 shows the EPN and LPP modulation observed for each individual case and behavior system presenting difference scalp maps (high–low arousal). A common scale was used (± 3 µV) to display the effects, which led to the truncation of the data in individual cases with large effects. The figure indicates a prototypical pattern of emotional modulation of the EPN and LPP in most of the participants. However, the magnitude of modulation varied across behavior systems and was on average larger for sexual reproduction and disease avoidance than for predator fear. Furthermore, each behavioral system showed a considerable variation between individuals, including displays of small to no effect in some cases.

**Fig. 4.**
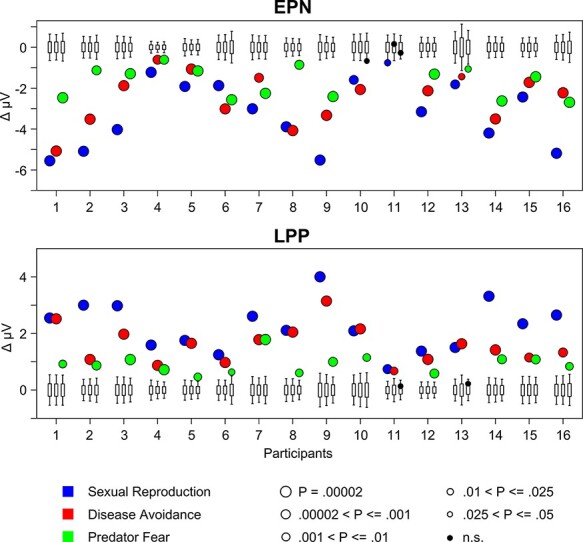
Outcome of the case-by-case statistics for the EPN and LPP components. A boxplot displays the bootstrap distribution for each case. The bottom and top edges of the box indicate the 25th and 75th percentiles, while the whiskers indicate the 5th and 95th percentiles. The dots indicate the de facto measured amplitude difference (high–low arousal). Dots outside the range indicated by the whiskers represent significant effects (*P* < 0.05), with size and color indicating different *P* levels.

The findings from bootstrap single-subject analysis are displayed in Figure 4, which shows the measured EPN and LPP difference, the observed *P*-level of significance and the random-resampling bootstrap distribution. Regarding the EPN, all 16 participants showed significantly larger EPN amplitudes for erotic compared to romantic images (100%). Furthermore, all but one participant showed a significant EPN modulation (94%) for disease avoidance. While the magnitude of the emotional modulation of the EPN was in general smaller for predator fear than for sexual reproduction and disease avoidance, the effect reached significance in 14 out of 16 cases (88%).

A similarly robust emotional modulation was observed for the LPP component. All 16 participants showed a significant effect for sexual reproduction (100%) and disease avoidance (100%). For predator fear, emotional modulation of the LPP was generally smaller in size compared to the two other behavior systems, with 14 out of 16 cases reaching significance (88%).

Tables 1 and 2 illustrate how different levels of *P*-criteria affect the proportion of significant cases for the EPN and LPP, respectively. Across all picture categories, the number of significant cases showed the expected decline with more stringent *P*-criteria. This decline reached significance for the strongest *P*-criterion compared to all four other *P*-criteria, EPN: *χ*^2^(4) = 29.1, *P* < 0.001, post hoc *P*_s_* *≤ 0.011, LPP: *χ*^2^(4) = 45.7, *P* < 0.001, post hoc *P*_s_ ≤ .033. Separate testing for the three behavior systems revealed that the effect was primarily driven by predator fear. Specifically, the decrease in the number of significant cases reached significance for the strictest *P*-criterion for the EPN compared to the *P* ≤ 0.05 and *P* ≤ 0.025 criteria (*χ*^2^(4) = 12.0, *P* = 0.017, post hoc *P*_s_ = 0.03) and for the LPP compared to the *P* ≤ 0.05, *P* ≤ 0.025 and *P* ≤ 0.01 criteria (*χ*^2^(4) = 33.3, *P* < 0.001, post hoc *P*_s_ < 0.001).

Comparing between behavior systems, no differences in sensitivity were observed for the EPN at any of the five *P*-level criteria (*χ*^2^(2)s ≤ 3.0, *P* ≥ 0.22). For the LPP, lower numbers of significant cases for predator fear compared to sexual reproduction and disease avoidance reached significance for the two strictest *P*-criteria (*P* < 0.001: *χ*^2^(2) = 14.3, *P* = 0.001, post hoc *P*_s_ < 0.007; *P* < 0.00002: *χ*^2^(2) = 20.7, *P* < 0.001, post hoc *P*_s_ ≤ 0.001).

### Specificity analysis

The specificity of the effect was determined by comparing left-right mirrored pictures for which no EPN/LPP effect was expected. Relying on a criterion of *P* < 0.05, a sizable number of false-positive tests emerged for the EPN (13% of test overall) as well as LPP (10% of test overall). As shown in Tables 1 and 2, the proportion of false alarms considerably decreased with stricter *P*-level criteria. For the EPN component, *χ*^2^(4) = 72.8, *P* < 0.001, post hoc testing indicated a significant decline from *P* < 0.05 to *P* < 0.025 criterion, *P* = 0.007. In addition, the *P* < 0.05 and *P* < 0.025 criteria had higher false alarm rates than the three stricter *P*-criteria, *P*_s_ ≤ .045. For the LPP component, *χ*^2^(4) = 56.2, *P* < 0.001, false alarm rate was also higher for the *P* < 0.05 criterion than the *P* < 0.025 criterion (*P* = 0.019) and the four other *P*-criteria, *P*_s_ ≤ 0.019. In addition, for the *P* < 0.025 criterion, false alarm rate was higher as compared to the *P* < 0.001 and *P* < 0.00002 criteria, *P*_s_ ≤ 0.019.

Separate analyses by behavior system revealed significant decreases of false alarm rates with stricter *P*-criteria for the EPN and LPP components for all three behavior systems, *χ*^2^(4) > 13.2, *P*_s_ ≤ 0.01. Higher false alarm rates were observed for the *P* < 0.05 criterion compared to the three strictest criteria (*P* < 0.01, *P* < 0.001 and *P* < 0.00002) for the EPN for all three behavior systems and for the LPP for sexual reproduction and predator fear (post hoc *P*_s_ ≤ 0.027). The LPP in the disease avoidance condition showed a smaller false alarm rate at the *P* < 0.05 level, reaching significance in the comparison of the *P* < 0.05 and *P* < 0.00002 criteria (*P *=* *0.019).

Similar false alarm rates were observed for the three behavioral systems (see Tables 1 and 2). False alarm rates did not differ as a function of behavior systems at any of the *P*-criteria for EPN nor LPP components, *χ*^2^(2) ≤ 2.0, *P*_s_ ≥ 0.368.

### Control analyses

#### Fixed time windows

In the main analysis, EPN and LPP were scored in automatically determined latency windows. To provide a more restrictive approach, a control analysis determined the single-case effects based on the identical time windows as used for the group analysis for the EPN and LPP components. Relying on the *P* < 0.05 criterion, the proportions of significant tests were identical in the fixed time window analysis for sexual reproduction (EPN: 100% and LPP: 100%) and disease avoidance (EPN: 100% and LPP: 100%) (EPN: 100%, LPP: 100%) as observed in the analyses based on individual time windows. For predator fear, the proportion of significant cases was the same for the EPN (88%) across both analyses. However, the proportion of significant cases was lower for the LPP in the fixed (75%) compared to the individual (88%) latency analyses.

**Table 1.** Proportion (%) of significant EPN effects associated with different *P*-level criteria when an effect is predicted, i.e. sensitivity, and when no effect is predicted, i.e. false alarm rate

	*P* < 0.05	*P* < 0.025	*P* < 0.01	*P* < 0.001	*P* < 0.00002
Sexual reproduction					
Sensitivity	100	100	94	94	81
False alarm rate	13	6	3	0	0
Disease avoidance					
Sensitivity	94	94	88	88	75
False alarm rate	14	6	0	0	0
Predator fear					
Sensitivity	88	88	81	81	63
False alarm rate	13	8	2	2	0

**Table 2.** Proportion (%) of significant LPP effects associated with different *b*-level criteria when an effect is predicted, i.e. sensitivity, and when no effect is predicted, i.e. false alarm rate

	*P* < 0.05	*P* < 0.025	*P* < 0.01	*P* < 0.001	*P* < 0.00002
Sexual reproduction					
Sensitivity	100	100	100	100	94
False alarm rate	13	5	3	0	0
Disease avoidance					
Sensitivity	100	100	100	94	81
False alarm rate	8	6	5	2	0
Predator fear					
Sensitivity	88	88	81	50	19
False alarm rate	13	6	3	0	0

#### Habituation analyses

Stimulus repetition may alter the sensitivity of the EPN and LPP components to assess emotional modulation effects. To assess habituation effects, for each individual case, trials were divided into first and second halves and submitted to bootstrap analyses. For the EPN component, the number of significant cases provided little evidence for emotional habituation effects, with slightly larger proportions of significant cases in the second half of the trials for sexual reproduction (first half: 94%, second half: 100%), disease avoidance (81%, 94%) and predator fear (81%, 94%). Furthermore, there was also little evidence for habituation effects for the LPP component, which showed similar proportions of significant cases for sexual reproduction (100%, 100%) and disease avoidance (100%, 94%) while the number of significant cases was lower in the second block for predator fear (81%, 63%). McNemar’s test revealed neither for the EPN (*P* = 0.063) nor the LPP (*P* = 0.219) a significant difference in the proportion of significant tests between the first and second halves of the trials.

## Discussion

A case-by-case approach was used to explore the emotional modulation of the EPN and LPP within the individual case in multiple behavior systems, namely sexual reproduction, disease avoidance and predator fear. Most tests were significant for behavior systems of sexual reproduction (EPN: 100% and LPP: 100%), disease avoidance (EPN: 94% and LPP: 100%) and predator fear (EPN: 88% and LPP: 88%). Overall, assessing neural correlates of affective stimulus evaluation at the level of the individual case appears to be promising with respect to subject generality and the generality across emotional behavior systems.

The current findings suggest that it is feasible to probe the process of affective stimulus evaluation within individuals with respect to multiple emotional stimulus categories. Specifically, the proportion of significant EPN and LPP tests for the three behavior systems observed here was similar to previous research in which these three behavior systems were probed separately, i.e. each case viewed pictures from one behavior system (Schupp and Kirmse, 2021). Noteworthy, 13 out of 16 cases showed significant EPN and LPP modulations for all three behavior systems providing within-subject replication of the effect. Probing the process of affective stimulus evaluation with respect to multiple emotional stimulus categories can accordingly make a strong case at the individual level for the presumed cause–effect relation that affective stimulus significance guides selective attention processes as revealed by the EPN and LPP components.

The occurrence of non-significant EPN and LPP effects challenges the common-to-all principle. In the absence of an objective standard for deciding whether a non-significant effect represents a true absent effect or a false negative, interpretation is ambiguous. This ambiguity is accentuated when only one test is available. Multiple tests can alleviate the problem by determining whether the absence of significant EPN and LPP effects in an individual case is content-specific or general and observed across multiple domains of emotional experience. In the present data, non-significant EPN and LPP effects were limited to a single behavior domain in three participants. One further participant had generally small ERP effects, only reaching significance for the EPN for erotic stimuli and for the LPP for erotica and mutilations. Thus, the few instances of non-significant EPN and LPP tests appear to be stimulus-specific rather than reflecting a general phenomenon across all three domains of behavior systems in an individual participant.

Furthermore, methodological issues need to be considered for the interpretation of non-significant cases. To assure a common-to-all effect and avoid the issue of approximate replication (Kapur *et al.*, 2012), we took a conservative approach in the main analyses by scoring EPN and LPP components in a priori defined sensor clusters and only adjusted time windows individually. In addition, an even more restrictive control analysis using pre-defined EPN and LPP time windows as used in the group analysis yielded highly similar results. Further analyses determined the effects of stimulus repetition by comparing findings from single-subject analyses based on separate tests from first and second halves of trials. Resonating with previous group research (Schupp *et al.*, 2006b; Ferrari *et al.*, 2020), no systematic decline of significant tests was observed as a function of stimulus repetition. Furthermore, even when based on only half the trials used in the main analysis, a high proportion of tests were significant for the EPN and LPP components, in particular for sexual reproduction. However, individual differences regarding brain anatomy may lead to EPN/LPP effects with a somewhat different topography in some individual cases. In exploratory analyses, we observed that relaxing the criteria related to the topography of the effect affects the outcome of some but not all non-significant tests. Furthermore, in some cases, EEG sensitivity may be too limited to reveal small effects. Future studies might therefore combine EEG measures with Magnetoencephalography (MEG) or functional imaging as complementary measures of brain activity to study affective stimulus evaluation (Keuper *et al.*, 2014; Frank and Sabatinelli, 2019).

While both the EPN and LPP components are presumed to provide insights into how stimuli we encounter in the world are continuously evaluated regarding their affective significance, they have been associated with different functional meanings. Specifically, two-stage models distinguish between a first large capacity perceptual scanning stage, which provides a more or less complete analysis of sensory information, and a second capacity-limited stage of processing, which enables conscious recognition and attentive processing (Öhman, 1979, 1986; Chun and Potter, 1995; Potter, 2012). Viewed from this perspective, larger EPN and LPP components can possibly be thought of as neural reflections of a processing advantage of high-arousing emotional stimuli with the EPN reflecting a call for processing resources and the LPP indicating access to second-stage processing (Schupp *et al.*, 2006a; Flaisch *et al.*, 2019). Our data suggest a negative relationship of the magnitude of the emotional modulation of the EPN and LPP. The exploratory analysis confirmed this impression by indicating significant correlations for sexual reproduction, *r* = −0.81, *P* = 0.002; disease avoidance, *r* = −0.52, *P* = 0.004; and predator fear, *r* = −0.46, *P* = 0.07. However, only in one case and specific to the predator fear domain, non-significant results converged for the EPN and LPP (see Figure 4). Furthermore, there is reason to assume that the EPN/LPP relationship depends on the experimental context in which the affective stimulus evaluation is examined. For instance, group research studying emotion processing in conflict paradigms and multiple stimulus conditions observed differential EPN and LPP effects (e.g. Ikeda *et al.*, 2013; Flaisch *et al.*, 2019). Future research may use the case-by-case approach to advance two-stage theories on the functional meaning of the EPN and LPP components.

The magnitude and statistical strength of the EPN and LPP effects across the three behavioral systems provided evidence for both general patterns and idiosyncratic variations. For instance, the magnitude of emotional modulations was larger for sexual reproduction than predator fear in 15 cases for the EPN and all 16 participants for the LPP. Furthermore, chi-square tests indicated the proportion of significant cases to be less stable for predator fear at stricter *P*-criteria. On the one hand, this may reflect that erotic stimuli are most potent in activating the appetitive system, leading to accentuated responses in somatic, autonomic and central nervous measures (Bradley *et al.*, 2001; Schupp *et al.*, 2004; Sabatinelli *et al.*, 2005). On the other hand, since the goal of optimizing experimental designs is critical for the case-by-case approach, future research is needed to improve the stimulus materials used as prototypical stimuli to engage the predator fear system (cf. Öhman *et al.*, 2000) and to develop stimulus materials tapping into further categories of emotional experience. Furthermore, responding to images related to disease avoidance showed idiosyncratic patterns with responses in some individuals close to or even exceeding responses to sexual reproduction, while other individuals showed less pronounced EPN and LPP modulations more akin to predator fear. However, explicit regulation of attention focus needs to be considered in future research when considering idiosyncratic variations to pictures related to disease avoidance, e.g. some participants may voluntarily withdraw attention from pictures showing mutilated bodies.

Beyond revealing an empirical regularity common to all, a case-by-case approach may contribute to the translation of basic research to the clinical domain by allowing inferences about individual cases. This reasoning is based on the Research Domain Criteria (RDoC) project’s aim of identifying new ways of classifying mental disorders in which the clustering of groups of individual patients is based on their individual response profiles (Cuthbert, 2014; Insel and Cuthbert, 2015) and on research that indicates selective attention deficits to specific stimuli in eating disorders, the anxiety spectrum and drug addiction (Mogg and Bradley, 1998; De Houwer *et al.*, 2004; Shafran *et al.*, 2007). Accordingly, studying affective stimulus evaluation across multiple categories of emotional experience may allow an individual’s emotional landscape to be explored, with a focus on exaggerated/blunted responses to selected stimulus contents. However, the trade-off between sensitivity and specificity as a function of *P*-criteria as well as behavioral system needs to be considered when evaluating the potential of the current research program to biomarker development (Woo and Wager, 2015; van der Miesen *et al.*, 2019). Specifically, while test sensitivity significantly declined when applying a very strict *P*-criterion (*P* < 0.00002), a substantial proportion of the tests was significant when relying on stricter *P*-criteria than *P* < 0.05, i.e. *P* < 0.01 and *P* < 0.001, particularly regarding the behavioral domains of sexual reproduction and disease avoidance. In addition, reliance on stronger *P*-criteria than *P* < 0.05 significantly reduced false alarms. Overall, future research needs to further refine the experimental protocol by allowing a reliance on a more stringent *P*-criterion than the conventional *P* < 0.05 to balance the trade-off between sensitivity and specificity as diagnostic criteria for biomarker development (Woo and Wager, 2015).
